# Creatininium hydrogen maleate

**DOI:** 10.1107/S1600536811040050

**Published:** 2011-10-12

**Authors:** A. Jahubar Ali, S. Athimoolam, S. Asath Bahadur

**Affiliations:** a Department of Science and Humanities, National College of Engineering, Maruthakulam, Tirunelveli 627 151, India; b Department of Physics, University College of Engineering Nagercoil, Anna University of Technology Tirunelveli, Nagercoil 629 004, India; c Department of Physics, Kalasalingam University, Anand Nagar, Krishnan Koil 626 190, India

## Abstract

In the title compound, C_4_H_8_N_3_O^+^·C_4_H_3_O_4_^−^, the cations and anions are linked through N—H⋯O hydrogen bonds making a ionic pair with an *R*_2_^2^(8) ring motif. These ionic pairs are further connected through another N—H⋯O hydrogen bond, leading to an *R*_6_^6^(16) ring motif around the inversion centres  of the unit cell. These approximately planar aggregates are further connected through weak van der Waals inter­actions in the unit cell. The anions have a characteristic intra­molecular O—H⋯O hydrogen bond with a self-associated ring *S*(7) motif.

## Related literature

For related structures, see: Ali *et al.* (2011 ▶); Bahadur, Kannan *et al.* (2007 ▶); Bahadur, Sivapragasam *et al.* (2007 ▶); Bahadur, Rajalakshmi *et al.* (2007 ▶). For hydrogen-bonding motif notation, see: Bernstein *et al.* (1995 ▶); Desiraju (1989 ▶). For the importance of creatinine, see: Madaras & Buck (1996 ▶); Sharma *et al.* (2004 ▶); Narayanan & Appleton (1980 ▶). 
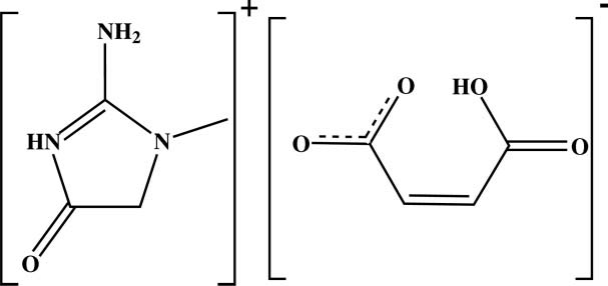


## Experimental

### 

#### Crystal data

C_4_H_8_N_3_O^+^·C_4_H_3_O_4_^−^
*M* *_r_* = 229.20
Monoclinic, 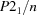

*a* = 5.6271 (4) Å
*b* = 24.8915 (17) Å
*c* = 7.7752 (6) Å
β = 108.69 (2)°
*V* = 1031.62 (18) Å^3^
*Z* = 4
Mo *K*α radiation
μ = 0.12 mm^−1^
*T* = 293 K
0.24 × 0.21 × 0.17 mm

#### Data collection

Bruker SMART APEX CCD area-detector diffractometer
9754 measured reflections
1823 independent reflections
1699 reflections with *I* > 2σ(*I*)
*R*_int_ = 0.022

#### Refinement

*R*[*F* ^2^ > 2σ(*F* ^2^)] = 0.038
*wR*(*F* ^2^) = 0.102
*S* = 1.06
1823 reflections
162 parameters
H atoms treated by a mixture of independent and constrained refinement
Δρ_max_ = 0.23 e Å^−3^
Δρ_min_ = −0.22 e Å^−3^

### 

Data collection: *SMART* (Bruker, 2001 ▶); cell refinement: *SAINT* (Bruker, 2001 ▶); data reduction: *SAINT*; program(s) used to solve structure: *SHELXTL/PC* (Sheldrick, 2008 ▶); program(s) used to refine structure: *SHELXTL/PC*; molecular graphics: *PLATON* (Spek, 2009 ▶); software used to prepare material for publication: *SHELXTL/PC*.

## Supplementary Material

Crystal structure: contains datablock(s) global, I. DOI: 10.1107/S1600536811040050/hg5104sup1.cif

Structure factors: contains datablock(s) I. DOI: 10.1107/S1600536811040050/hg5104Isup2.hkl

Supplementary material file. DOI: 10.1107/S1600536811040050/hg5104Isup3.cml

Additional supplementary materials:  crystallographic information; 3D view; checkCIF report

## Figures and Tables

**Table 1:** Hydrogen-bond geometry (Å, °)

*D*—H⋯*A*	*D*—H	H⋯*A*	*D*⋯*A*	*D*—H⋯*A*
N14—H14⋯O22	0.93 (2)	1.79 (2)	2.725 (2)	178 (2)
N16—H15*A*⋯O24^i^	0.89 (2)	1.96 (2)	2.833 (2)	168 (2)
N16—H15*B*⋯O21	0.93 (2)	1.88 (2)	2.804 (2)	174 (2)
O23—H23*A*⋯O21	1.00 (3)	1.46 (3)	2.457 (2)	174 (2)

Symmetry code: (i) 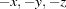.

**Fig. 1.**
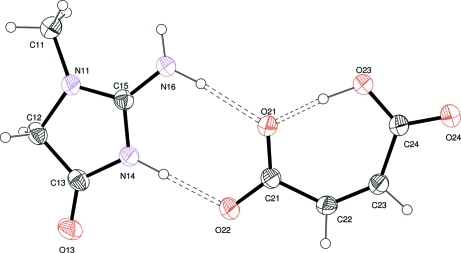
The molecular structure of the title compound (I) with the numbering scheme for the atoms and 50% probability displacement ellipsoids. H bonds are drawn as dashed lines.

**Fig. 2.**
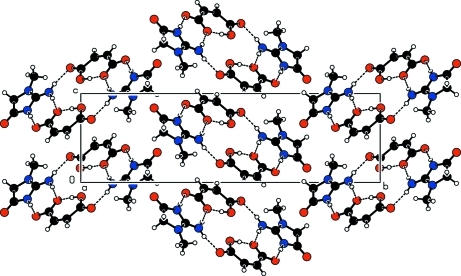
Packing diagram of the molecules viewed down the b-axis. H atoms not involved in the H-bonds (dashed lines) are omitted for clarity.

## supplementary crystallographic information

### Comment

Intermolecular forces play an essential role in the formation of supramolecular systems which are useful for definite social applications. In which, the phenomenon of hydrogen bond has its importance in the areas of molecular recognition, crystal engineering research and supramolecular chemistry. Their strength and directionality is responsible for crystal packing and entire molecular arrays (Desiraju, 1989). We are interested on the the specificity of recognition between inorganic / organic acids and cretinine molecule. Creatinine, a blood metabolite of considerable importance in clinical chemistry, particularly as an indicator of renal function. It has been proven that determination of creatinine is more valuable for the detection of renal dysfunction than that of urea (Sharma *et al.*, 2004). In renal physiology, creatinine clearance (CCr; Madaras & Buck, 1996) is the volume of blood plasma that is cleared of creatinine per unit time. Clinically, creatinine clearance is a useful measure for estimating the glomerular Filtration rate (GFR) of the kidneys. Anabnormal level of creatinine in biological fluids is an indicator of various disease states (Narayanan & Appleton, 1980).

The asymmetric part of the title compound, (I), contains one creatininium cation and one maleate anion (Fig.1). The protonation of the N site of the cation is evident from C—N bond distances and the other bond distances and angles are comaparable with Creatininium cinnamate (Ali *et al.*, 2011), Creatininium hydrogen oxalate monohydrate (Bahadur, Kannan *et al.*, 2007), Creatininium benzoate (Bahadur, Sivapragasam *et al.*, 2007) and bis(creatininium) sulfate (Bahadur, Rajalakshmi *et al.*, 2007). The deprotonation on the one of the –COOH groups of the maleic acid is confirmed from that –COO^-^ bond geometry.

In the crystal structure, the molecular aggregations are stabilized through a two dimensional hydrogen bonding pattern (Fig. 2; Table 1). Cations are linked to anions forming an ion pair through two N—H···O bonds that produce ring *R*_2_^2^(8) motifs (Bernstein *et al.*, 1995). The same type of ring motif is observed in previously reported structures from our laboratory. Anions are having a characteristic intramolecular O—H···O hydrogen bond with a self-associated S(7) motif. This cation-anion pairs are further linked through another N—H···O hydrogen bond leading to a ring *R*_6_^6^(16) motif around the inversion centres of the unit cell. This ring motifs are almost planar. These ring motifs are connected through weak Van der Waals interactions in the unit cell.

### Experimental

The title compound was crystallized from an aqueous mixture containing creatinine and maleic acid in the stoichiometric ratio of 1:1 at room temperature by slow evaporation technique.

### Refinement

All the H atoms except the atoms involved in hydrogen bonds were positioned geometrically and refined using a riding model, with C—H = 0.93 (–CH) and 0.96 Å (–CH_3_) and *U*_iso_(H) = 1.2–1.5 *U*_eq_ (parent atom). H atoms involved in hydrogen bonds were located from differential fourier map and refined isotropically.

### Crystal data

**Table:**

C_4_H_8_N_3_O^+^·C_4_H_3_O_4_^−^	*F*(000) = 480
*M**_r_* = 229.20	*D*_x_ = 1.476 Mg m^−^^3^
Monoclinic, *P*2_1_/*n*	Mo *K*α radiation, λ = 0.71073 Å
Hall symbol: -P 2yn	Cell parameters from 3738 reflections
*a* = 5.6271 (4) Å	θ = 2.3–24.6°
*b* = 24.8915 (17) Å	µ = 0.12 mm^−^^1^
*c* = 7.7752 (6) Å	*T* = 293 K
β = 108.69 (2)°	Block, colourless
*V* = 1031.62 (18) Å^3^	0.24 × 0.21 × 0.17 mm
*Z* = 4	

### Data collection

**Table:**

Bruker SMART APEX CCD area-detector diffractometer	1699 reflections with *I* > 2σ(*I*)
Radiation source: fine-focus sealed tube	*R*_int_ = 0.022
graphite	θ_max_ = 25.0°, θ_min_ = 2.9°
ω scans	*h* = −6→6
9754 measured reflections	*k* = −29→29
1823 independent reflections	*l* = −9→9

### Refinement

**Table:**

Refinement on *F*^2^	Primary atom site location: structure-invariant direct methods
Least-squares matrix: full	Secondary atom site location: difference Fourier map
*R*[*F*^2^ > 2σ(*F*^2^)] = 0.038	Hydrogen site location: inferred from neighbouring sites
*wR*(*F*^2^) = 0.102	H atoms treated by a mixture of independent and constrained refinement
*S* = 1.06	*w* = 1/[σ^2^(*F*_o_^2^) + (0.0593*P*)^2^ + 0.2134*P*] where *P* = (*F*_o_^2^ + 2*F*_c_^2^)/3
1823 reflections	(Δ/σ)_max_ < 0.001
162 parameters	Δρ_max_ = 0.23 e Å^−^^3^
0 restraints	Δρ_min_ = −0.22 e Å^−^^3^

### Special details

**Table:**

Geometry. All e.s.d.'s (except the e.s.d. in the dihedral angle between two l.s. planes) are estimated using the full covariance matrix. The cell e.s.d.'s are taken into account individually in the estimation of e.s.d.'s in distances, angles and torsion angles; correlations between e.s.d.'s in cell parameters are only used when they are defined by crystal symmetry. An approximate (isotropic) treatment of cell e.s.d.'s is used for estimating e.s.d.'s involving l.s. planes.
Refinement. Refinement of *F*^2^ against ALL reflections. The weighted *R*-factor *wR* and goodness of fit *S* are based on *F*^2^, conventional *R*-factors *R* are based on *F*, with *F* set to zero for negative *F*^2^. The threshold expression of *F*^2^ > σ(*F*^2^) is used only for calculating *R*-factors(gt) *etc*. and is not relevant to the choice of reflections for refinement. *R*-factors based on *F*^2^ are statistically about twice as large as those based on *F*, and *R*- factors based on ALL data will be even larger.

### Fractional atomic coordinates and isotropic or equivalent isotropic displacement parameters (Å^2^)

**Table:**

	*x*	*y*	*z*	*U*_iso_*/*U*_eq_	
C11	0.7258 (3)	0.15606 (7)	−0.1940 (2)	0.0572 (4)	
H11A	0.7728	0.1188	−0.1812	0.086*	
H11B	0.8598	0.1769	−0.2113	0.086*	
H11C	0.5775	0.1604	−0.2970	0.086*	
N11	0.6762 (2)	0.17415 (4)	−0.03149 (16)	0.0437 (3)	
C12	0.7738 (3)	0.22340 (5)	0.0666 (2)	0.0459 (3)	
H12A	0.7215	0.2547	−0.0107	0.055*	
H12B	0.9555	0.2227	0.1152	0.055*	
C13	0.6576 (3)	0.22303 (5)	0.2162 (2)	0.0444 (3)	
O13	0.6882 (2)	0.25506 (4)	0.33827 (17)	0.0623 (3)	
N14	0.5068 (2)	0.17843 (4)	0.18824 (16)	0.0418 (3)	
C15	0.5203 (2)	0.15060 (5)	0.04068 (18)	0.0390 (3)	
N16	0.3960 (2)	0.10634 (5)	−0.01514 (19)	0.0489 (3)	
H14	0.403 (3)	0.1680 (7)	0.255 (2)	0.056 (5)*	
H15A	0.412 (3)	0.0883 (8)	−0.109 (3)	0.062 (5)*	
H15B	0.296 (3)	0.0947 (7)	0.052 (2)	0.060 (5)*	
O21	0.1034 (2)	0.07770 (4)	0.20235 (16)	0.0606 (3)	
O22	0.1978 (2)	0.14924 (4)	0.37969 (15)	0.0555 (3)	
C21	0.0833 (3)	0.10606 (5)	0.33167 (19)	0.0428 (3)	
C22	−0.0877 (3)	0.08869 (6)	0.43292 (19)	0.0451 (3)	
H22	−0.0881	0.1112	0.5284	0.054*	
C23	−0.2406 (3)	0.04642 (6)	0.41017 (19)	0.0450 (3)	
H23	−0.3351	0.0453	0.4887	0.054*	
C24	−0.2848 (3)	0.00087 (5)	0.28009 (18)	0.0433 (3)	
O23	−0.1681 (2)	−0.00175 (4)	0.16102 (15)	0.0601 (3)	
O24	−0.4294 (2)	−0.03477 (4)	0.28982 (16)	0.0573 (3)	
H23A	−0.056 (5)	0.0298 (11)	0.170 (3)	0.104 (8)*	

### Atomic displacement parameters (Å^2^)

**Table:**

	*U*^11^	*U*^22^	*U*^33^	*U*^12^	*U*^13^	*U*^23^
C11	0.0641 (10)	0.0581 (10)	0.0588 (9)	−0.0113 (7)	0.0330 (8)	−0.0079 (7)
N11	0.0461 (6)	0.0370 (6)	0.0523 (7)	−0.0057 (5)	0.0220 (5)	−0.0044 (5)
C12	0.0446 (7)	0.0338 (7)	0.0586 (8)	−0.0044 (5)	0.0157 (6)	−0.0021 (6)
C13	0.0428 (7)	0.0332 (7)	0.0550 (8)	0.0017 (5)	0.0126 (6)	−0.0044 (6)
O13	0.0661 (7)	0.0493 (7)	0.0743 (7)	−0.0085 (5)	0.0263 (6)	−0.0232 (6)
N14	0.0470 (6)	0.0343 (6)	0.0467 (6)	−0.0021 (5)	0.0188 (5)	−0.0025 (5)
C15	0.0404 (7)	0.0329 (7)	0.0439 (7)	0.0016 (5)	0.0139 (5)	0.0006 (5)
N16	0.0579 (8)	0.0412 (7)	0.0543 (7)	−0.0131 (5)	0.0274 (6)	−0.0098 (6)
O21	0.0827 (8)	0.0500 (6)	0.0664 (7)	−0.0229 (6)	0.0479 (6)	−0.0165 (5)
O22	0.0700 (7)	0.0399 (6)	0.0656 (7)	−0.0154 (5)	0.0345 (6)	−0.0100 (5)
C21	0.0505 (8)	0.0344 (7)	0.0458 (7)	−0.0009 (6)	0.0187 (6)	0.0002 (5)
C22	0.0579 (8)	0.0377 (7)	0.0452 (7)	−0.0016 (6)	0.0242 (6)	−0.0058 (6)
C23	0.0527 (8)	0.0413 (7)	0.0479 (7)	−0.0026 (6)	0.0255 (6)	−0.0015 (6)
C24	0.0495 (8)	0.0378 (7)	0.0455 (8)	−0.0041 (6)	0.0191 (6)	0.0000 (6)
O23	0.0839 (8)	0.0481 (6)	0.0638 (7)	−0.0241 (6)	0.0452 (6)	−0.0178 (5)
O24	0.0671 (7)	0.0485 (6)	0.0655 (7)	−0.0187 (5)	0.0340 (6)	−0.0107 (5)

### Geometric parameters (Å, °)

**Table:**

C11—N11	1.450 (2)	C15—N16	1.3025 (18)
C11—H11A	0.9600	N16—H15A	0.89 (2)
C11—H11B	0.9600	N16—H15B	0.93 (2)
C11—H11C	0.9600	O21—C21	1.2630 (17)
N11—C15	1.3206 (18)	O22—C21	1.2471 (17)
N11—C12	1.4552 (17)	C21—C22	1.490 (2)
C12—C13	1.506 (2)	C22—C23	1.334 (2)
C12—H12A	0.9700	C22—H22	0.9300
C12—H12B	0.9700	C23—C24	1.4862 (19)
C13—O13	1.2090 (17)	C23—H23	0.9300
C13—N14	1.3719 (17)	C24—O24	1.2225 (17)
N14—C15	1.3631 (18)	C24—O23	1.2967 (17)
N14—H14	0.934 (19)	O23—H23A	1.00 (3)
			
N11—C11—H11A	109.5	C13—N14—H14	127.1 (11)
N11—C11—H11B	109.5	N16—C15—N11	126.35 (13)
H11A—C11—H11B	109.5	N16—C15—N14	122.80 (13)
N11—C11—H11C	109.5	N11—C15—N14	110.84 (12)
H11A—C11—H11C	109.5	C15—N16—H15A	121.2 (12)
H11B—C11—H11C	109.5	C15—N16—H15B	115.6 (11)
C15—N11—C11	124.88 (12)	H15A—N16—H15B	123.2 (16)
C15—N11—C12	109.99 (11)	O22—C21—O21	123.41 (13)
C11—N11—C12	124.93 (12)	O22—C21—C22	116.82 (12)
N11—C12—C13	102.44 (11)	O21—C21—C22	119.76 (12)
N11—C12—H12A	111.3	C23—C22—C21	131.07 (13)
C13—C12—H12A	111.3	C23—C22—H22	114.5
N11—C12—H12B	111.3	C21—C22—H22	114.5
C13—C12—H12B	111.3	C22—C23—C24	130.69 (13)
H12A—C12—H12B	109.2	C22—C23—H23	114.7
O13—C13—N14	125.67 (14)	C24—C23—H23	114.7
O13—C13—C12	127.94 (13)	O24—C24—O23	120.46 (13)
N14—C13—C12	106.40 (11)	O24—C24—C23	118.75 (12)
C15—N14—C13	110.25 (12)	O23—C24—C23	120.77 (12)
C15—N14—H14	122.6 (11)	C24—O23—H23A	111.2 (14)
			
C15—N11—C12—C13	2.95 (15)	C12—N11—C15—N14	−2.40 (16)
C11—N11—C12—C13	178.00 (14)	C13—N14—C15—N16	179.61 (13)
N11—C12—C13—O13	177.46 (14)	C13—N14—C15—N11	0.69 (16)
N11—C12—C13—N14	−2.45 (14)	O22—C21—C22—C23	177.12 (16)
O13—C13—N14—C15	−178.69 (14)	O21—C21—C22—C23	−1.9 (2)
C12—C13—N14—C15	1.22 (15)	C21—C22—C23—C24	2.8 (3)
C11—N11—C15—N16	3.7 (2)	C22—C23—C24—O24	177.17 (16)
C12—N11—C15—N16	178.73 (13)	C22—C23—C24—O23	−1.3 (2)
C11—N11—C15—N14	−177.45 (14)		

### Hydrogen-bond geometry (Å, °)

**Table:**

*D*—H···*A*	*D*—H	H···*A*	*D*···*A*	*D*—H···*A*
N14—H14···O22	0.93 (2)	1.79 (2)	2.725 (2)	178 (2)
N16—H15A···O24^i^	0.89 (2)	1.96 (2)	2.833 (2)	168 (2)
N16—H15B···O21	0.93 (2)	1.88 (2)	2.804 (2)	174 (2)
O23—H23A···O21	1.00 (3)	1.46 (3)	2.457 (2)	174 (2)

Symmetry codes: (i) −*x*, −*y*, −*z*.
